# Self-Templated Highly Porous Gold Electrodes for Antibiofouling Electrochemical (Bio)Sensors

**DOI:** 10.3390/nano16020087

**Published:** 2026-01-08

**Authors:** Anisa Degjoni, Cristina Tortolini, Daniele Passeri, Andrea Lenzi, Riccarda Antiochia

**Affiliations:** 1 Department of Experimental Medicine, Sapienza University of Rome, V.le Regina Elena 324, 00161 Rome, Italy; anisa.degjoni@uniroma1.it (A.D.); cristina.tortolini@uniroma1.it (C.T.); andrea.lenzi@uniroma1.it (A.L.); 2 Department of Basic and Applied Sciences for Engineering, Sapienza University of Rome, Via A. Scarpa 14, 00161 Rome, Italy; daniele.passeri@uniroma1.it; 3 Research Center for Nanotechnology Applied to Engineering of Sapienza University of Rome (CNIS), P. le A. Moro 5, 00185 Rome, Italy

**Keywords:** highly porous gold, antibiofouling, self-templated electrodeposition, gold screen-printed electrode, bovine serum albumin, mass transport

## Abstract

Biofouling arises from non-specific adsorption of several components present in complex biofluids, such as full blood, on the surface of electrochemical biosensors, with a resulting loss of functionality. Most biomarkers of clinical relevance are present in biological fluids at extremely low concentrations, making antibiofouling strategies necessary in electrochemical biosensing. Here, we demonstrate the effect of a highly porous gold (h-PG) film electrodeposited on a gold screen-printed electrode (AuSPE) using a self-templated method via hydrogen bubbling as an antibiofouling strategy in electrochemical biosensor development following exposure of the electrode to bovine serum albumin (BSA) at two different concentrations (2 and 32 mg/mL). The h-PG film has a high electrochemically active surface area, 88 times higher than the AuSPE electrode, with a pore size ranging from 2 to 50 μm. A rapid decrease in the Faradaic current was observed with the unmodified AuSPE, attesting to the strong biofouling effect of BSA at both concentrations tested. Notably, the h-PG-modified electrode showed an initial peak current decline, more evident at a higher BSA concentration, followed by rapid electrode regeneration when the electrode was left idle in the biofouling solution. Similar results were obtained for unmodified and modified electrodes in real serum and plasma samples. The regeneration process, explained in terms of balance between h-PG pore size and protein size, the nanoscale architecture of the h-PG electrodes, and repulsive electrostatic forces, indicates the huge potential of the h-PG film for use in biomedical electrochemical sensing.

## 1. Introduction

Electrochemical biosensors represent a fundamental class of analytical devices demonstrating immense utility in clinical diagnostics, environmental monitoring, and continuous health surveillance [1,2,3,4]. These sensors capitalize on the inherent sensitivity and portability of electrochemical techniques, which rely on redox reactions to generate measurable output signals through cyclic voltammetry (CV), differential pulse voltammetry (DPV), or electrochemical impedance spectroscopy (EIS) [5]. Particularly, gold electrodes are frequently utilized as working elements due to gold’s chemical inertness and stability within the physiological potential window. Despite significant achievements, enabling these devices to operate reliably within complex biological matrices remains a critical engineering and chemical challenge [6,7].

The performance and long-term stability of electrochemical biosensors are fundamentally compromised by a ubiquitous phenomenon known as biofouling [8]. Biofouling refers to the non-specific accumulation of different organic molecules, including proteins, lipids, polysaccharides, and nucleotides, onto the electrode surface. This process is particularly pronounced in biological environments, where a vast array of species competes for adsorption sites. The consequences of biofouling are as follows: (i) a notable decrease in sensitivity; (ii) obstruction of mass transfer pathways; (iii) inhibition of electron transfer (ET) between the electrode and the target analyte; (iv) necessity of frequent biosensor recalibration; and (v) eventual complete loss of functionality [9,10]. For instance, implanted devices can rapidly develop a layer of accumulated biomolecules, potentially forming a fibrous capsule that physically impedes the diffusion of analytes toward the electrode surface, rendering the device inoperable post-implantation. Accordingly, the design of interfaces that effectively combine high analytical sensitivity with robust antifouling capabilities is essential for expanding the practical applicability of electrochemical biosensing systems, especially those destined for in vivo or long-term continuous operation [11,12,13,14,15,16].

In response to the pervasive problem of biofouling, researchers have historically developed several strategies aimed at either attenuating or outright inhibiting the non-specific adsorption of interfering species, particularly large proteins. These efforts can broadly be categorized into chemical modification techniques and strategies involving size-selective exclusion or topography modification.

A popular solution involves the chemical modification of electrode surfaces through the coating of ordered organic layers, such as Self-Assembled Monolayers (SAMs) [17,18,19,20,21]. The function of such layers is to act as a physical or chemical barrier against protein adsorption while maintaining electron transfer capability to the underlying electrode.

Poly (ethylene glycol) (PEG) is widely recognized as the “gold standard” of antibiofouling polymers due to its inherent non-toxicity and high hydrophilicity [22]. PEG functions primarily by creating a highly hydrated layer at the interface; when proteins approach, the layer’s compression generates a steric hindrance that leads to biomolecule repulsion [23,24]. Furthermore, grafting non-conductive, long-chain PEG can lead to the formation of layers exhibiting high impedance, consequently decreasing the electrode’s overall sensitivity.

An alternative, increasingly promising paradigm shifts the focus from chemically modifying the surface to manipulating the intrinsic nanostructure of the electrode itself to structurally mitigate biofouling effects. Nanoporous gold (NPG), also referenced as porous gold (PG), is an interesting material in this context, offering a high surface-to-volume ratio, catalytic properties, and inherent antibiofouling features [25,26,27]. Porous electrodes with larger features, such as macroporous gold (pore diameter > 1200 nm) and hierarchical gold (1200/60 nm bimodal pore network), show better performance than planar gold but still eventually succumb to passivation [25]. NPG electrodes, particularly those with pore sizes < 1200 nm, show the most robust antifouling performance [25].

Reduced graphene oxide (rGO) has also been successfully used as an antifouling matrix in electrochemical sensing. Ingber et al. [28] demonstrated that glutaraldehyde (GA) cross-linked bovine serum albumin (BSA) with aminated rGO or pentaamine-modified graphene nanoflake showed great potential as an antifouling electrode coating [29,30]. The BSA/rGOx/GA nanocomposite confirmed its antifouling properties when the coated electrodes were exposed to serum and whole blood, showing no significant changes in current density after 60 min incubation.

In this work, the antibiofouling properties of a self-templated electrodeposited h-PG-modified screen-printed gold electrode were investigated in physiological concentrations of albumin and in human serum and plasma. Other works reported the antibiofouling activity of h-PG fabricated according to the classical dealloying treatment but, to the best of our knowledge, this is the very first time that an electrochemically generated h-PG film was investigated for its antifouling properties. A current decrease was initially observed, but a total regeneration of the peak current was achieved after 45 min incubation in 2 and 32 mg/mL BSA and in real serum and plasma samples. A detailed comparison of the results obtained with h-PG with those obtained with classical dealloying treatments is also reported.

## 2. Materials and Methods

### 2.1. Reagents

Lyophilized bovine serum albumin (BSA, ≥98%), ammonium chloride (NH_4_Cl, 99.998%), tetrachloroauric (III) acid (HAuCl_4_, 99%), sodium phosphate monobasic (Na_2_HPO_4_, ≥99%), sodium phosphate dibasic (NaH_2_PO_4_, ≥99%), potassium chloride (KCl, 99–100.5%), potassium ferricyanide (III) (K_3_[Fe(CN)_6_], 99.0%), and potassium ferrocyanide (II) (K_4_[Fe(CN)_6_] 98.5–102.0%), were purchased from Merck (Merck Life Science S.r.l. Milan, Italy). Flat gold screen-printed electrodes (AuSPEs) (220AT) were purchased from Metrohm (Metrohm Italiana S.r.l., Oreggio, Italy).

All solutions were prepared in phosphate-buffered saline (PBS) containing 0.1 M phosphate buffer and 0.1 M KCl at pH 7.0. All experiments were conducted using high-purity deionized Milli-Q water (resistance: 18.2 MΩ cm at 25 °C; TOC < 10 µg/L) (Millipore, Molsheim, France).

### 2.2. Instrumentation

Scanning electron microscopy (SEM) was used to investigate the morphology of bare and modified electrodes, employing a scanning electron microscope (Tescan, Brno, Czech Republic).

Cyclic voltammetry (CV) and electrochemical impedance spectroscopy (EIS) measurements were carried out using an Autolab Potentiostat/Galvanostat (Eco Chemie, Utrecht, The Netherlands) featuring Nova 2.1 software (Eco Chemie, The Netherlands).

The experiments were conducted on SPEs consisting of a gold AT working electrode (high-temperature curing AT ink; d = 0.4 cm; Metrohm Italiana, Origgio, Italy), a silver reference electrode, and a gold AT auxiliary electrode.

The high-resolution images of AuSPE and h-PG/AuSPE in the absence and presence of 32 mg/mL BSA were acquired via digital microscopy (HIROX HRX-02 3D Digital Microscope, Limonest, France).

### 2.3. Modification of SPEs

The self-templated highly porous gold (h-PG) methodology was already realized in our laboratory by Bollella et al. [31,32,33]. Briefly, the two-step electrodeposition method was utilized as follows: (i) SPE electrodes were immersed in an electrochemical glass cell with 20 mL of solution containing 10 mM HAuCl_4_ and 2.4 mM NH_4_Cl, and electrodeposition was performed by cycling the potential between +0.8 and 0 V versus Ag/AgCl_sat_ for 25 scans at 50 mV/s; (ii) a fixed potential of −2 V versus Ag/AgCl_sat_ was applied for 60 s for the self-templated pore formation due to intense hydrogen bubbling. In terms of electrochemical characteristics, electroactive surface area (A_EA_) and roughness factor (ρ) were determined in 0.1 M KCl with 2 mM [Fe(CN)_6_]^3−/4−^, prepared in 10 mM PBS (pH = 7.0).

### 2.4. Electrochemical Measurements

The AuSPE and h-PG/AuSPEs electrodes were initially electrochemically characterized using the CV and EIS techniques. Measurements were performed by drop-casting 100 µL 0.1 M KCl solution with 2 mM [Fe(CN)_6_]^3−/4−^ in 0.1 mM PBS (redox probe solution) onto the electrode surface. For BSA measurements, 10 µL of BSA (dissolved in 0.1 mM PBS) was added to the same solution, at final concentrations of 2 mg/mL and 32 mg/mL, respectively. The antibiofouling properties of AuSPE and h-PG/AuSPE sensors after BSA addition were evaluated by CV at an applied potential range of −0.3–+0.5 V, versus Ag/AgCl, and EIS measurements over a frequency range of 0.1–10^5^ Hz, using an AC amplitude of 10 mV and under open-circuit potential (OCP) conditions.

### 2.5. Real Samples Analysis

For serum and plasma collection, blood samples were withdrawn from a healthy volunteer. To obtain the serum, blood was allowed to clot at room temperature for 15 min. Following, the clot was removed via refrigerated centrifugation at 1000× *g* for 10 min.

For plasma collection, blood was withdrawn in an anticoagulant ethylenediaminetetraacetic acid (EDTA)-coated tube and immediately centrifugated via refrigerated centrifugation at 1000× *g* for 10 min.

Both serum and plasma samples were used immediately after collection and stored at 4 °C while handling. They were drop-casted onto the electrode surface (100 μL) and underwent CV measurements over a time range of 0–60 min.

## 3. Results 

### 3.1. Electrochemical Response of AuSPE and h-PG/AuSPE in Biofouling Conditions

The electrochemical properties of AuSPE before and after h-PG electrodeposition were investigated using CV and EIS in the absence and presence of BSA, a homolog of, but a cheaper molecule than, human serum albumin (HSA), the most abundant protein in blood, known as the main biofouling agent when detecting analytes in blood. BSA has a globular shape (14 × 4 nm) and a molecular weight of about 66 kDa, with typical human blood serum concentrations ranging between 35 and 50 mg/mL. BSA is known in the literature to adsorb on gold electrodes [34,35,36] by showing, in the presence of a redox probe, (i) an increase in peak-to-peak separation (∆*E*_p_) and a decrease in the peak Faradic current in cyclic voltammograms and (ii) an increase in charge transfer resistance (R_CT_) in Nyquists plots by the EIS technique, clearly demonstrating that BSA adsorption on the surface of a gold electrode hinders the electron transfer of the redox probe. By exposing the electrode surface to different concentrations of BSA, we aimed to simulate the degree of biofouling that would be expected in clinically relevant samples, such as plasma and serum.

#### 3.1.1. Cyclic Voltammetry Studies

To study the antibiofouling properties following h-PG modification, CVs were performed in 2 mM [Fe(CN)_6_]^3−/4−^ in 0.1 mM PBS, continuously for up to 60 min, in the presence of two different BSA concentrations, 2 mg/mL and 32 mg/mL. These two concentration values were chosen in order to investigate the effect of BSA in a large of range of concentrations up to its physiological levels in human serum (32 mg/mL). Any fouling of the electrode surface would result in an increased degree of irreversibility of the voltametric curve, due to slow electron transfer kinetics, with a decrease in peak current and increase in ∆*E*_p_, as explained above.

**Figure 1:**
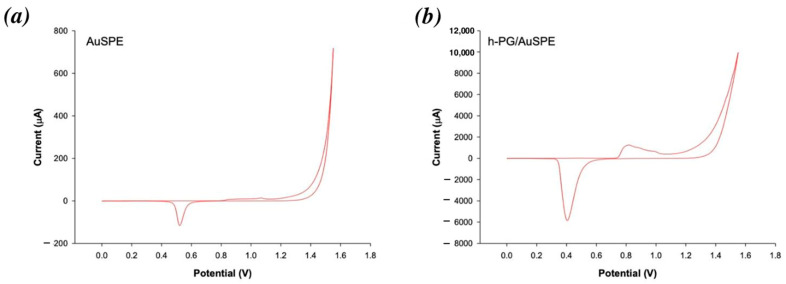
CVs of AuSPE (**a**) and h-PG/AuSPE (**b**) carried out in 0.1 M H_2_SO_4_ at scan rate of 100 mV/s.

The real surface area of the h-PG electrodes was calculated by CV in 0.1 M H_2_SO_4_ at a scan rate of 100 mV/s, integrating the charge required to reduce the gold oxide formed during the positive sweep. By assuming a charge density for the reduction of gold oxide of 390 µC cm^−2^, a value of 56.2 cm^2^ was obtained for the real surface area of the h-PG electrode, a value which is about 88 times higher compared to bare electrode (0.64 cm^2^) as a consequence of the network of nanoporosities (Figure 1).

The heterogeneous electron transfer rate constants (k_0_, cm s^−1^) for AuSPE before and after the modification with h-PG were calculated with a method proposed by Lavagnini et al. that merges the Klingler–Kochi and Nicholson and Shain methods developed for irreversible and reversible systems, respectively [37,38]. The h-PG electrode showed a k_0_ value (k_0_ = 6.4 ± 0.5 × 10^3^ cm s^−1^) three times higher than the unmodified electrode (k_0_ = 2.1 ± 0.4 × 10^3^ cm s^−1^), attesting to the faster electron transfer kinetics (Table 1).

**Figure 2:**
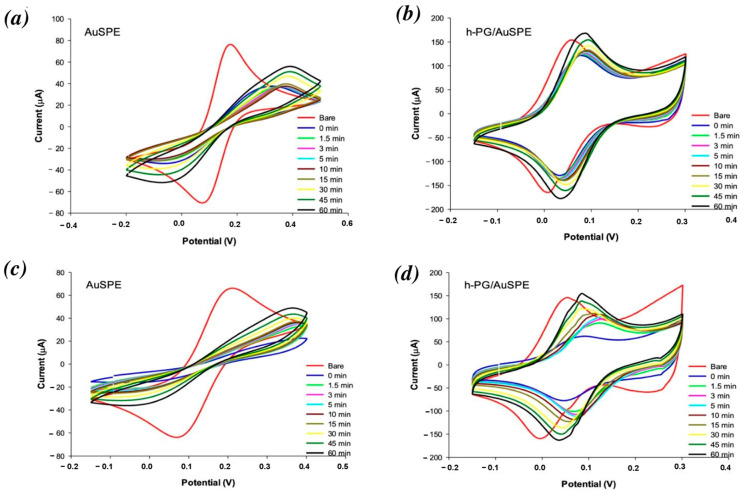
CVs of AuSPE (**a**,**c**) and h-PG/AuSPE (**b**,**d**) before (red curves) and after incubation in BSA concentrations of 2 mg/mL (**a**,**b**) and 32 mg/mL (**c**,**d**) for different amounts of time: 0 min (blue curve); 1.5 min (green curve); 3 min (pink curve); 5 min (cyan curve); 10 min (brown curve); 15 min (green curve); 30 min (yellow curve); 45 min (dark green curve); and 60 min (black curve). Redox probe: 2 mM [Fe(CN)_6_]^3−/4−^ containing 0.1 M KCl solution, in 0.1 M PBS, pH = 7.0; scan rate: 100 mV/s.

Figure 2 shows the CVs of the bare AuSPE (a and c) and h-NP/AuSPE (b and d) recorded before (red curves) and after incubation in BSA at 2 mg/mL (a and b) and 32 mg/mL (c and d) in a time range of 0–60 min (curves of different colors). Two well-defined redox peaks attesting to the diffusion-controlled, reversible behavior of the redox probe were obtained with unmodified and modified electrodes before BSA addition (Figure 2, red curves). Given that [Fe(CN)_6_]^3−/4−^ is a reversible redox probe with fast one-electron transfer kinetics, the ideal peak-to-peak separation is 59 mV. A Δ*E*_p_ value of 49 mV was obtained with the h-PG electrode, compared to 92 mV for the bare electrode, indicating a significant improvement in the h-PG electrode, with a surface prone to fast electron transfer. The anodic and cathodic peak currents show a remarkable enhancement as a consequence of the increase in the real surface area (Figure 2b,d), compared to unmodified AuSPE (Figure 2a,c). However, the enhancement is much lower if compared to the increase in real active surface area. This fact can be ascribed to the fast electron transfer kinetics of [Fe(CN)_6_]^3−/4−^ in solution, which does not benefit from the large area effect. In the case of fast electron transfer reactions, the concentration of the oxidized mediator at the outermost pore layer rapidly drops to zero, leading to a zero concentration in the inner pores, with a consequent non-use of the inner pore surface.

After BSA addition, the unmodified electrode shows flatter CV curves with progressive peak current decreases and ∆E_p_ increases at increasing incubation times, typical of a total irreversible behavior. The drastic signal decreases in the Faradic peak current after the initial BSA addition at 2 and 32 mg/mL were 62% and 95%, respectively (Figure 2a,c, blue curves). These represent the normalized values calculated by the formula (*i* − *i_bare_*)/*i_bare_* × 100%, where *i* represents the peak current at different times and *i_bare_* the peak current baseline before BSA addition. Such a strong effect is due to the lower electron transfer kinetics caused by BSA adsorption on the electrode surface. The current remains at the minimal value for about 15 min; then, a slow recovery of the peak current obtained before BSA addition is observed at both BSA concentrations (Figure 2a,c, yellow, dark green and black curves), although the initial current is not reached during the 60 min observation (Table 2). The term “electrode regeneration” will be used in the rest of this article to refer to the recovery of the electrochemical parameters and of peak current and impedance values, referring to CV and EIS experiments, respectively, registered before BSA addition (initial conditions).

The increasing degree of protein absorption at increasing incubation times is also demonstrated by the increased peak potential separation values, more evident at higher BSA concentrations, with ∆*E*_p_ values ranging from 90 mV (before addition of BSA) to 320 and 373 mV at 2 and 32 mg/mL, respectively, after 60 min incubation in BSA (Figure 2a,c, black curves, Table 2).

On the contrary, the h-PG electrode allowed for a quasi-reversible process after BSA addition (Figure 2b,d). With 2 mg/mL BSA addition, the peak current showed an immediate slight current reduction while maintaining a similar ∆*E*_p_, with a slight positive potential shift (Figure 2b, blue curve). However, progressive peak current increases are observed over time, starting at 1.5 min (Figure 2b, green curve) and reaching a total peak current regeneration after 45 min (Figure 2b, dark green curve).

Similar behavior was observed when a higher BSA concentration was added (32 mg/mL). Once again, after an initial peak current decrease, progressive increases in peak current over time (Figure 2d) were registered, showing 100% peak current regeneration after 45 min (Figure 2d, dark green curve).

In order to better highlight the recovery rates of the modified electrodes after the addition of BSA at the two different concentrations, plots of peak current vs. time are reported (Figure S1). It is interesting to note that the slopes of the curves in the range of 0–30 min are 0.93 μA/min at 2 mg/mL and 2.79 μA/min at 32 mg/mL (Figure S1, red curves), whereas between 30 and 60 min, the two slopes are comparable, attesting to a three times higher recovery rate with the lower BSA concentration in the first 30 min and similar recovery rates after 30 min.

No significant ∆*E*_p_ increases were noticed with h-PG/AuSPE, even in the presence of 32 mg/mL BSA, with ∆*E*_p_ values ranging between 49 and 41 mV before and after 60 min incubation in BSA. All these results attest to the excellent antibiofouling properties of the h-PG electrodes compared to AuSPE (Table 2).

The comparison of anodic peak current and peak-to-peak separation recorded over time obtained with unmodified and modified AuSPE after BSA addition at 2 and 32 mg/mL is reported in Table 2.

The following electrochemical parameters are summarized in Table 1 for AuSPE and the h-PG-modified electrode: anodic peak current (I_pa_), peak-to-peak separation (Δ*E*_p_), electroactive area (A_EA_), roughness factor (ρ), and heterogeneous rate constant (k_0_). A_EA_ was calculated from the slope of the I_p_ vs. v^1/2^ plot, and the value was used in the Randles–Sevcik equation:

I_p_ = 2.686 × 10^5^ n^3/2^ A_e_ D_0_^1/2^ C_0_ v^1/2^ (1)

where I_p_ = voltametric peak current (A), n = number of electrons, A_EA_ = electroactive area (cm^2^), D_0_ = diffusion coefficient (7.6 × 10^−6^ cm^2^ s^−1^ for ferricyanide), C_0_ = concentration (mol cm^−3^), v = scan rate (V s^−1^), and ρ = ratio of electroactive to geometric area.

**Figure 3:**
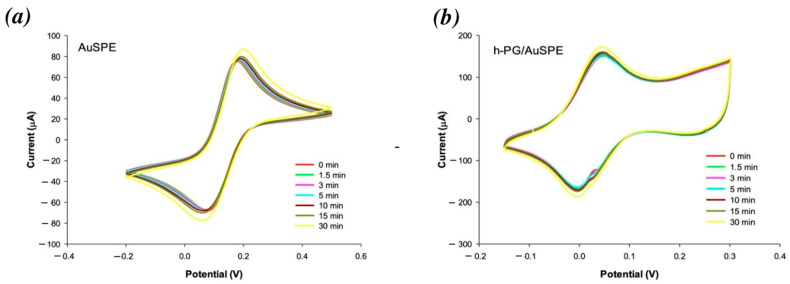
CVs of (**a**) AuSPE and (**b**) h-PG/AuSPE in absence of BSA recorded at the following interval times: 0 min (red curve); 1.5 min (green curve); 3 min (pink curve); 5 min (cyan curve); 10 min (brown curve); 15 min (dark green curve); and 30 min (yellow curve). Redox probe: 2 mM [Fe(CN)_6_]^3−/4−^ containing 0.1 M KCl solution, pH = 7.0, in 0.1 M PBS. Scan rate: 100 mV/s.

To confirm that the observed current changes were due to effective BSA adsorption, control measurements were carried out over time with the AuSPE and h-PG-modified electrodes in the same redox probe solution without addition of BSA (Figure 3a,b). In both cases, no peak current decrease was observed in CV measurements over 30 min (yellow curves), confirming the role of BSA in modifying the gold electrode surface.

#### 3.1.2. Electrochemical Impedance Spectroscopy Studies

**Figure 4:**
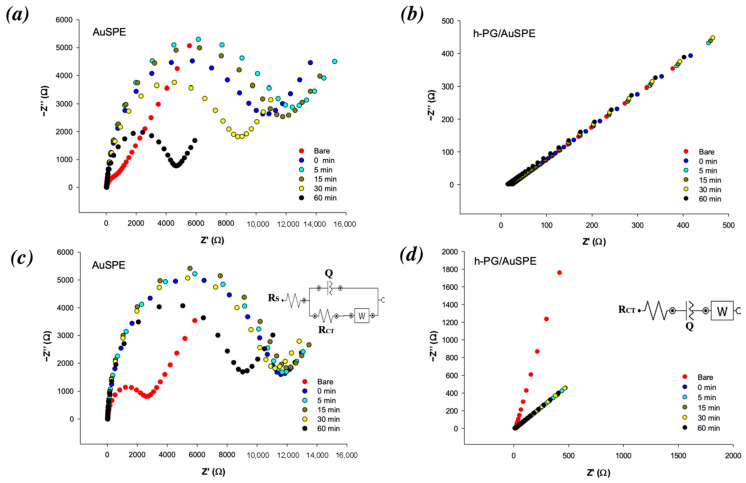
Nyquist plots of AuSPE and h-PG/AuSPE before BSA addition (red curves, “bare” electrodes) and after incubation in different BSA concentrations, 2 mg/mL (**a**,**b**) and 32 mg/mL (**c**,**d**), for different amounts of time: 0 min (blue curve); 5 min (cyan curve); 15 min (brown curve); 30 min (yellow curve); and 60 min (black curve). Redox probe: 2 mM [Fe(CN)_6_]^3−/4−^ containing 0.1 M KCl in 0.1 M PBS. Frequency range: 0.1–10^5^ Hz, with an AC amplitude of 10 mV. Inset: [R(Q[RW])] circuit was used for fitting the experimental data for AuSPE, and [RQW] circuit was used for fitting the experimental data for h-PG AuSPE.

EIS was employed to explore the biofouling features of AuSPE and the h-PG-modified electrode before and after incubation in BSA. Figure 4 presents the Nyquist plots for bare (a and c) and h-PG AuSPE (b and d) using [Fe(CN)_6_]^3−/4−^ as a redox probe, without BSA (red curves) and after incubation in BSA at 2 mg/mL (a and c) and 32 mg/mL (b and d), for different amounts of time (5, 15, 30 and 60 min). In the high-frequency region, Nyquist plots exhibited semicircle diameters, which corresponds to the charge transfer resistance (R_CT_), which is hindered in biofouling conditions, leading to an increase in the semicircle diameter. At lower frequencies, the impedance is governed by a diffusion-limited process. The impedance spectra obtained with AuSPEs were fitted using the simple Randles circuit [R(Q[RW])] (Figure 4c, inset), whereas the [RQW] circuit was employed for fitting of h-PG AuSPEs (Figure 4d, inset).

All fitted impedance values are reported in Table 3 and Table 4. The R_CT_ values of the modified electrode significantly declined from 2350 Ω (unmodified electrode) to 19.6 Ω, highlighting the large enhancement in sensor conductivity due to h-PG modification, consistent with the strong peak current increases obtained in the CV experiments, as previously reported (Figure 2, red curves).

It is interesting to note that after an initial increase in R_CT_ values after BSA addition on both electrodes (blue and cyan curves compared to red curves in Figure 4), a decrease in impedance is observed in all plots with time, which corresponds to the recovery of the peak current, as seen in the CV curves in Figure 2. The recovery is 100% after 30 min for h-PG-modified electrodes at both BSA concentrations tested, as evaluated from the data reported in Table 4. Similar results were obtained with CV curves, as shown in Figure 2b,d, with a 100% peak current recovery reached after 45 min (dark green curves, compared to black curves). For unmodified electrodes, the electrode “regeneration”, in terms of the recovery of the initial impedance values, in particular R_CT_ values, is not completely obtained, as clearly shown in the Nyquist plots in Figure 4a,c, where the curves registered after 60 min (black curves) show semicircle diameters lower than those obtained after immediate addition of BSA (blue curves) but still definitely larger than those registered before addition of BSA (red curves). These results are in perfect agreement with those obtained with CV experiments, where the curves after 60 min (Figure 2a,c, black curves) show peak current values lower than those obtained before BSA addition (red curves).

### 3.2. SEM Characterization

**Figure 5:**
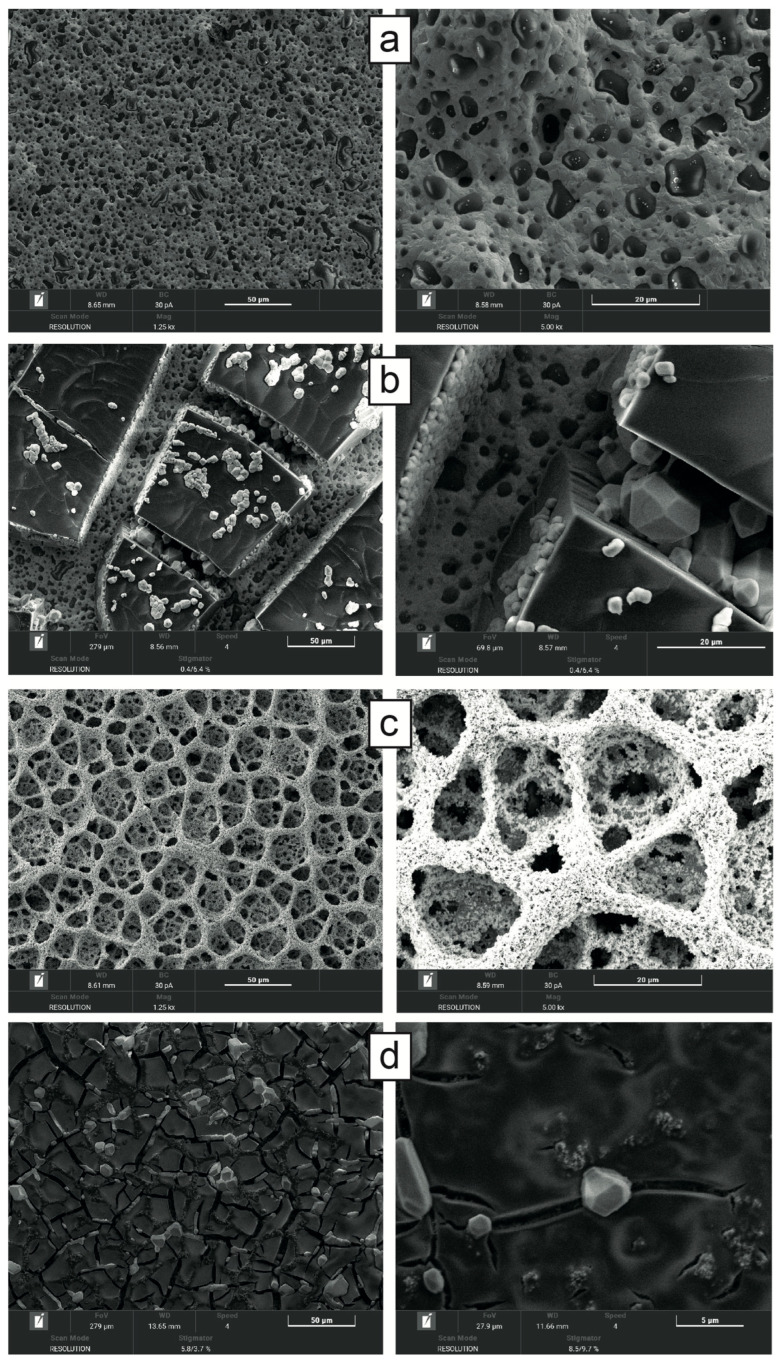
SEM images of AuSPE (**a**,**b**) and h-PG-AuSPE (**c**,**d**) at two different magnifications before (**a**,**c**) and after (**b**,**d**) the addition of 32 mg/mL BSA at 30 min incubation time.

Figure 5 demonstrates the results of the morphological characterization of AuSPE and h-PG/AuSPE before and after the addition of 32 mg/mL BSA using the SEM technique, which provides information on the size, distribution, and shape of the tested materials.

The AuSPE before surface modification shows a smooth surface (Figure 5a). After the electro polymerization process, interconnected three-dimensional porous structures were obtained (Figure 5c). The resulting h-NG electrode presented a 3D foam-like structure with a wide pore size distribution ranging from 5 to 20 μm, in accordance with data reported in the literature for similar structures of microporous electrodes [39,40]. The pore size distribution is reported in Figure S2. This large pore size distribution is important for the electrochemical reactivity observed, as most pores are large enough to allow for the free movement of the redox probe (<1 nm) deep into the porous gold structure of the electrode.

After BSA addition, a heterogeneous, highly non-porous thick structure is evident on the unmodified AuSPE (Figure 5b), responsible for the hindrance of the probe electron transfer. A thin, porous layer is clearly visible on modified h-PG electrode (Figure 5d). BSA molecules may enter the pores because of their smaller dimensions, with the formation of a porous layer adsorbed both in the outer and inner part of the h-PG structure. In particular, Figure 5d shows the porous BSA layer covering the outer structure. The porous nature of this layer allows the redox probe to move towards the electrode surface without suppressing the current level and associated signal change.

Low-magnification images showed the presence of microscopic cracking on the membrane surface. The cracks may result from temperature changes, humidity, and swelling during incubation time. These results are supported by acquisition of high-resolution images of the above-mentioned electrodes via digital microscopy and are reported in Figure S3.

### 3.3. Discussion

The antibiofouling effect of the gold electrode surface is a balance between several factors: (i) mass transport limitations due to the pore size of the h-PG-modified AuSPE, compared to the molecular size of BSA (about 14 × 4 nm) [30]; (ii) electrostatic repulsions between the h-PG electrode and BSA, both negatively charged at pH = 7 of the measurement solution; (iii) protein and salt concentrations; and (iv) ionic strength of the medium.

According to the first factor, it is possible to distinguish three cases: (i) The median pore radius of h-PG is smaller than the size of BSA: the nanostructured surface of h-PG/AuSPE may act as a sieve, allowing transport into the deeper pores of ([Fe(CN)_6_]^3−/4−^), which has a hydrodynamic radius <1 nm, while blocking the BSA. (ii) The median pore radius of h-PG is comparable to the size of BSA: BSA molecules may adsorb onto the top of the electrode surface without penetrating into the deeper pores, still allowing for the electron transfer of the redox probe at the inner pore surface. (iii) The median pore radius of h-PG is larger than the size of BSA: BSA molecules penetrate into the pores and adsorb onto the inner pores surface with a total hindrance of the electron transfer. A total adsorption of BSA on the electrode surface is also obtained with unmodified gold electrodes after exposure to BSA [25].

On the basis of this hypothesis, it is possible to explain the results reported by Patel et al. [25], where the passivation in presence of the fouling agent was directly proportional to the pore size. In particular, microporous gold (1200 nm) showed a rapid current drop, attesting electrode passivation, whereas nanoporous gold (5–50 nm pores) did not passivate after exposure to BSA 2 mg/mL for 60 min.

Different results were reported by Saraf et al. [9], where a nanoporous (25 nm) gold electrode showed an immediate peak current reduction with 25 mg/mL BSA concentrations, up to 23% of its initial value after 45 min incubation [9,41]. However, a regeneration of the peak current was progressively observed with time when the electrode was left idle in the fouling solution. The regeneration process was quite slow, as the electrode took 50 h to gain 96% of its initial current value. The authors hypothesize that a BSA layer is adsorbed in the inner nanopores but it still allows for a slow diffusion of the redox mediator towards the electrode surface. The total regeneration of the peak current is due to the saturation of the nanopores with the redox mediator achieved during the idle time.

Similar behavior was observed in our study (Figure 2), although the degradation and successive peak current regeneration occurred more rapidly, with a current increase observed after 1.5 min incubation and a total regeneration achieved after 45 min. These results can be ascribed to the different nanoarchitecture of the electrodeposited h-NP gold film compared to the NP film formed by dealloying, which may allow a rapid formation of a porous thin BSA layer, resulting in a faster diffusion of the redox probe inside the porous structure.

In general, h-PG-modified gold electrodes show a marked improvement in retaining electrochemical activity relative to the diffusion of a redox probe compared to bare electrodes, thus showing antifouling properties. If pores are smaller than 5–10 nm, mass transport restrictions hinder BSA or other large proteins from reaching the inner pore surface while still allowing them access to the small redox probe molecules. In the case of h-PG films with larger pore dimensions, the porosity and nanoscale architecture (curvature) of the h-PG electrodes render the blocking of the surface by protein adsorption and/or unfolding less effective, still allowing a good electron transfer of the redox probe.

Another possible explanation for the antifouling behavior of h-PG electrodes relates to the electrical double layer nano-exclusion phenomenon reported by Saraf and co-workers [9]. According to this hypothesis, the electrical double layer generated within the nanopores could impart an additional force that could repel anionic BSA molecules from adsorbing to the anionic h-PG surface. The isoelectric points (pI) are 4.7 and 5.2 for BSA and gold, respectively, which are both negatively charged in the measurement solution (pH = 7) and therefore electrostatically repulse each other [42].

On the contrary, the rapid decline in peak current observed in CV curves for unmodified AuNPEs clearly indicated a non-porous adsorbed BSA layer. This hypothesis is confirmed by the SEM images, where a non-porous layer of BSA is clearly visible immediately after addition of 32 mg/mL BSA. On the contrary, on the unmodified AuSPE, a thick non-porous BSA layer is present after addition of 32 mg/mL BSA, attesting to total passivation of the electrode surface.

### 3.4. Real Sample Analysis

**Figure 6:**
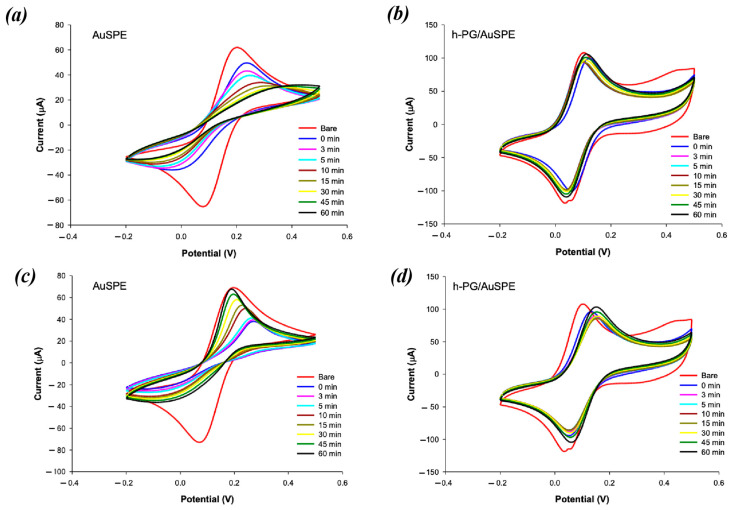
CVs of AuSPE (**a**,**c**) and h-PG/AuSPE (**b**,**d**) in 0.1 M PBS before (red curves, “bare electrodes”) and after drop-casting human serum (**a**,**b**) and human plasma (**c**,**d**) registered after 0 min (blue curve); 3 min (pink curve); 5 min (cyan curve); 10 min (brown curve); 15 min (green curve); 30 min (yellow curve); 45 min (dark green curve); and 60 min (black curve). Redox probe: 2 mM [Fe(CN)_6_]^3−/4−^ containing 0.1 M KCl solution in 0.1 M PBS, pH = 7.0; scan rate: 100 mV/s.

In order to evaluate the performance of the electrodeposited h-PG/AuSPE compared to the unmodified electrode in a more complex biological matrix, one sample of serum and one sample of blood from a healthy volunteer were tested by cyclic voltammetry over a period of 0–60 min with 2 mM ferro/ferri as the redox probe. The results are reported in Figure 6. Very little change in the shape of the voltammograms was registered for h-PG/AuSPE in both serum (Figure 6b) and plasma (Figure 6d), with a small current decrease observed only in plasma, probably because of the presence of fibrinogen, another biofouling agent present in plasma. On the contrary, for unmodified AuSPE, the voltametric curves became flatter in both serum and plasma, attesting to an irreversible behavior (Figure 6a,c). The results clearly show that the h-PG-modified electrode is able to retain its redox activity in both serum and plasma, while it is clearly diminished in the unmodified AuSPE.

## 4. Conclusions

The development of reliable electrochemical biosensors for deployment in complex biological samples hinges on overcoming electrode biofouling. Here, we demonstrated the excellent antibiofouling properties of h-PG-modified gold electrodes, another advantage besides their high electrode surface area and fast kinetics.

Nanoporous gold is known to integrate enhanced electrochemical performance with structural antifouling resistance, based on size exclusion and favorable electrostatic interactions compared to unmodified gold electrodes. However, all h-PG electrodes investigated for antifouling properties up to now have been developed by classical dealloying methods. In our work, a porous gold film was formed by an electrodeposition method via a hydrogen bubble template, which shows several advantages: small amount of gold required, speed, no harsh reagents involved, and ease of use. The excellent antifouling properties exhibited by the h-PG electrodes are not the result of a single mechanism and may arise from a combination of physical exclusion and electrostatic repulsion exclusion. In particular, the following three aspects must be considered: (i) mass transport limitations; (ii) the porosity and nanoscale architecture of h-PG electrodes, which make surface blocking by protein adsorption and/or unfolding less successful; and (iii) an additional repulsive electrostatic force between the negatively charged BSA molecules and the anionic h-PG surface generated by the electrical double layer within the pores.

The results obtained in real samples demonstrated that h-PG is highly effective at suppressing non-specific adsorption of major proteins like HSA and fibrinogen, present in human plasma, maintaining signal integrity even after long-term exposure (60 min) to complex biofluids, like human serum and plasma.

The excellent results obtained with self-templated h-PG electrodes offer significant potential for miniaturization and integration into next-generation marketable biomedical devices, such as wearable biosensors for long-term continuous monitoring systems. Further studies will be carried out in order to explore structure–property relationships and better elucidate whether one of the proposed mechanisms or a combination of them was responsible for the excellent antifouling properties of the h-PG electrodes in order to further enhance anti-fouling strategies for diverse biological applications.

## Figures and Tables

**Table 1:** Anodic peak current (I_pa_), peak-to-peak separation (ΔE_p_), electroactive area (A_EA_), roughness factor (ρ), and heterogeneous rate constant (k_0_) of AuSPE and h-PG/AuSPE electrodes. All values for AuSPE and h-PG/AuSPE electrodes are the mean values of Figure 2a,c and Figure 2b,d, respectively. Geometric area = 0.11 cm^2^.

Electrode	I_pa_ (µA)	ΔE_p_ (V)	A_EA_/(cm^2^)	*ρ*	k_0_/10^−3^ cm s^−1^
AuSPE	66.8 ± 5.3	90.3 ± 8.7	0.040 ± 0.001	0.4	2.1 ± 0.4
h-PG/AuSPE	150.2 ± 13.8	48.8 ± 3.7	0.150 ± 0.002	1.4	6.4 ± 0.5

**Table 2:** Anodic peak current (I_pa_) and peak-to-peak separation (ΔE_p_) of AuSPE and h-PG/AuSPE electrodes after BSA addition over time. All values reported are the means of three replicates (n = 3).

Time (min)	I_pa_ (µA)	ΔE_p_ (V)	I_pa_ (µA)	ΔE_p_ (V)
	AuSPE + BSA 2 mg/mL		h-PG/AuSPE + BSA 2 mg/mL	
0	25.2 ± 1.3	258.8 ± 17.6	110.5 ± 8.3	49.1 ± 3.2
15	19.2 ± 0.9	344.2 ± 25.4	120.3 ± 6.1	49.5 ± 2.8
30	19.6 ± 0.8	341.8 ± 19.6	138.6 ±10.8	49.5 ± 3.8
45	24.5 ± 0.9	336.9 ± 20.3	159.2 ± 11.4	48.9 ± 3.4
60	26.3 ± 1.6	319.5 ± 18.2	168.5 ± 8.9	48.9 ± 2.9
	AuSPE + BSA 32 mg/mL		h-PG/AuSPE + BSA 32 mg/mL	
0	3.2 ± 0.2	293.0 ± 13.8	51.6 ± 2.9	27.2 ± 1.9
15	5.3 ± 0.6	385.7 ± 18.3	100.2 ± 4.6	38.7 ± 2.5
30	7.4 ± 0.2	388.2 ± 21.2	135.4 ± 10.7	38.9 ± 2.7
45	9.2 ± 0.3	380.9 ± 14.3	146.7 ± 8.2	39.1 ± 2.5
60	10.2 ± 0.6	373.5 ± 19.7	154.3 ± 9.8	49.5 ± 2.8

**Table 3:** Fitting parameters for AuSPE before and after addition of BSA obtained using the simple Randles circuit [R(Q[RW])]. All values reported are the means of three replicates (n = 3).

Time (min)	R_S_ (Ω)	R_CT_ (kΩ)	Q (µΩ^−1^·s^N^)	N	W (µΩ^−1^·s^1/2^)
	AuSPE				
	26.0 ± 1.3	2.35 ± 0.12	1.86 ± 0.07	0.960 ± 0.030	398 ± 9.95
	AuSPE + BSA 2 mg/mL				
0	37.8 ± 0.7	9.6 ± 0.5	2.38 ± 0.12	0.931 ± 0.05	211 ± 10.8
5	38.3 ± 1.4	11.2 ± 0.09	2.28 ± 0.09	0.932 ± 0.09	212 ± 7.4
15	35.6 ± 1.2	10.7 ± 0.7	2.23 ± 0.14	0.931 ± 0.08	240 ± 11.6
30	31.4 ± 1.8	8.1 ± 0.2	2.25 ± 0.12	0.928 ± 0.05	298 ± 16.8
60	24.9 ± 1.5	4.3 ± 0.1	2.15 ± 0.08	0.933 ± 0.07	544 ± 25.9
	AuSPE + BSA 32 mg/mL				
0	29.1 ± 2.1	10.8 ± 0.5	1.56 ± 0.02	0.945 ± 0.03	402 ± 28.3
5	28.6 ± 1.3	10.9 ± 0.7	1.67 ± 0.05	0.960 ± 0.08	398 ± 20.6
15	27.9 ± 0.8	11.0 ± 0.5	1.68 ± 0.12	0.972 ± 0.04	360 ± 23.8
30	26.2 ± 1.2	10.2 ± 0.8	1.69 ± 0.08	0.976 ± 0.09	340 ± 31.4
60	22.2 ±1.5	8.22 ± 0.4	1.71 ± 0.1	0.981 ± 0.06	308 ± 19.7

**Table 4:** Fitting parameters for h-PG/AuSPE before and after addition of BSA obtained using the [RQW] circuit. All values reported are the means of three replicates (n = 3).

Time (min)	R_CT_ (Ω)	Q (µΩ^−1^·s^N^)	N
	h-PG/AuSPE		
	19.6 ± 1.2	822 ± 43.7	0.853 ±0.032
	h-PG/AuSPE + BSA 2 mg/mL		
0	30.7 ± 1.8	514 ± 32.3	0.851 ± 0.047
5	31.7 ± 1.6	448 ± 25.8	0.842 ± 0.039
15	29.3 ± 2.1	407 ± 30.5	0.837 ± 0.054
30	20.9 ± 1.3	431 ± 33.8	0.835 ± 0.064
60	19.7 ± 1.4	433 ± 26.3	0.826 ± 0.031
	h-PG/AuSPE + BSA 32 mg/mL		
0	24.3 ± 1.1	465 ± 25.9	0.859 ± 0.043
5	23.4 ± 0.8	424 ± 31.6	0.847 ± 0.022
15	21.7 ± 1.3	377 ± 19.7	0.812 ± 0.029
30	19.6 ± 0.9	376 ± 21.8	0.824 ± 0.038
60	15.1 ± 0.8	362 ± 24.3	0.819 ± 0.027

## Data Availability

The original contributions presented in this study are included in the article/Supplementary Materials. Further inquiries can be directed to the corresponding authors.

## Supplementary Materials

The following supporting information can be downloaded at https://www.mdpi.com/article/10.3390/nano16020087/s1. Figure S1. Histograms showing peak current vs. time of AuSPE and h-PG/AuSPE in presence of BSA; Figure S2. Histograms pore size distribution; Figure S3. Digital microscopy images of working electrode surfaces.
