# Supplementary material for: Self-Templated Highly Porous Gold Electrodes for Antibiofouling Electrochemical (Bio)Sensors
Source: Nanomaterials (Basel). 2026 Jan 8;16(2):87. doi: 10.3390/nano16020087 (PMC12844270; doi:10.3390/nano16020087)
Supplement: Supplementary file 1 [file nanomaterials-16-00087-s001.zip › nanomaterials-4039007-supplementary.pdf]

Supplementary Materials

Self-Templated Highly Porous Gold Electrodes for Antibiofouling Electrochemical (Bio)sensors

Anisa Degjoni [1], Cristina Tortolini [1], Daniele Passeri [2,3], Andrea Lenzi [1] and Riccarda Antiochia [1,*]

[1] Department of Experimental Medicine, Sapienza University of Rome, V. le Regina Elena 324, 00161 Rome, Italy; anisa.degjoni@uniroma1.it (A.D.); cristina.tortolini@uniroma1.it (C.T.); andrea.lenzi@uniroma1.it (A.L.)
[2] Department of Basic and Applied Sciences for Engineering, Sapienza University of Rome, Via A. Scarpa 14, 00161 Rome, Italy; daniele.passeri@uniroma1.it
[3] Research Center for Nanotechnology Applied to Engineering of Sapienza University of Rome (CNIS), P. le A. Moro 5, 0 0185 Rome, Italy
* Correspondence: riccarda.antiochia@uniroma1.it

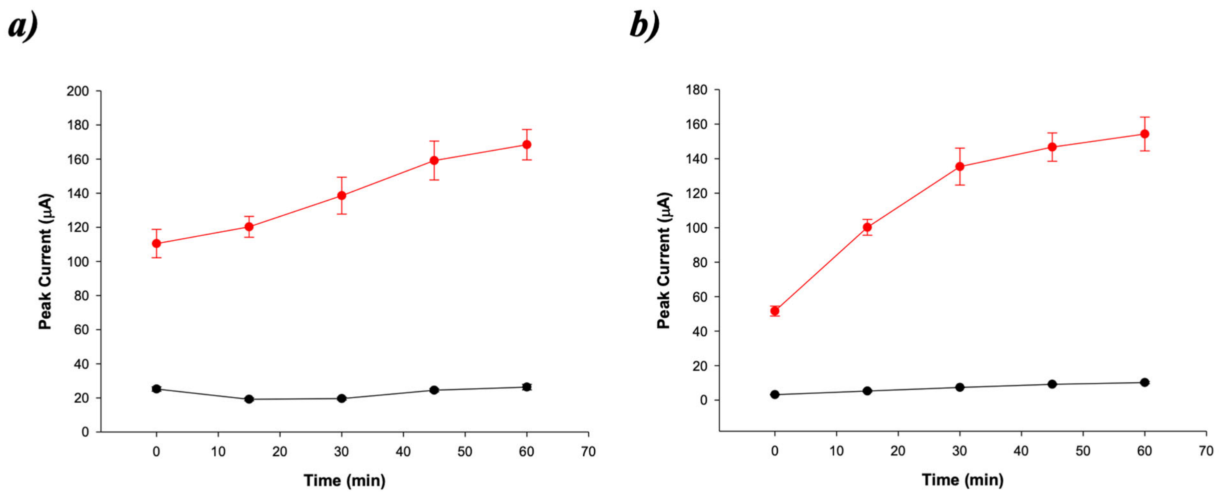

Figure S1. Histograms showing peak current vs. time of AuSPE (black histograms) and h-PG/AuSPE (red histograms) in presence of BSA: (a) 2 mg/mL; (b) 32 mg/mL.
Time 0 minutes= right after BSA addition.

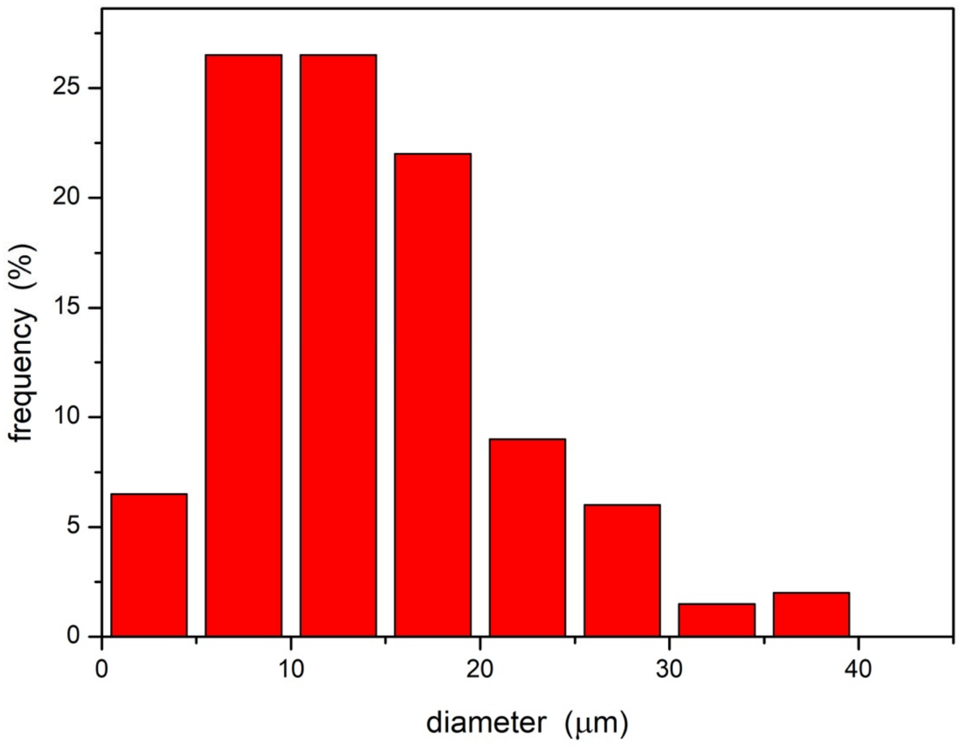

Figure S2. Histograms pore size distribution.

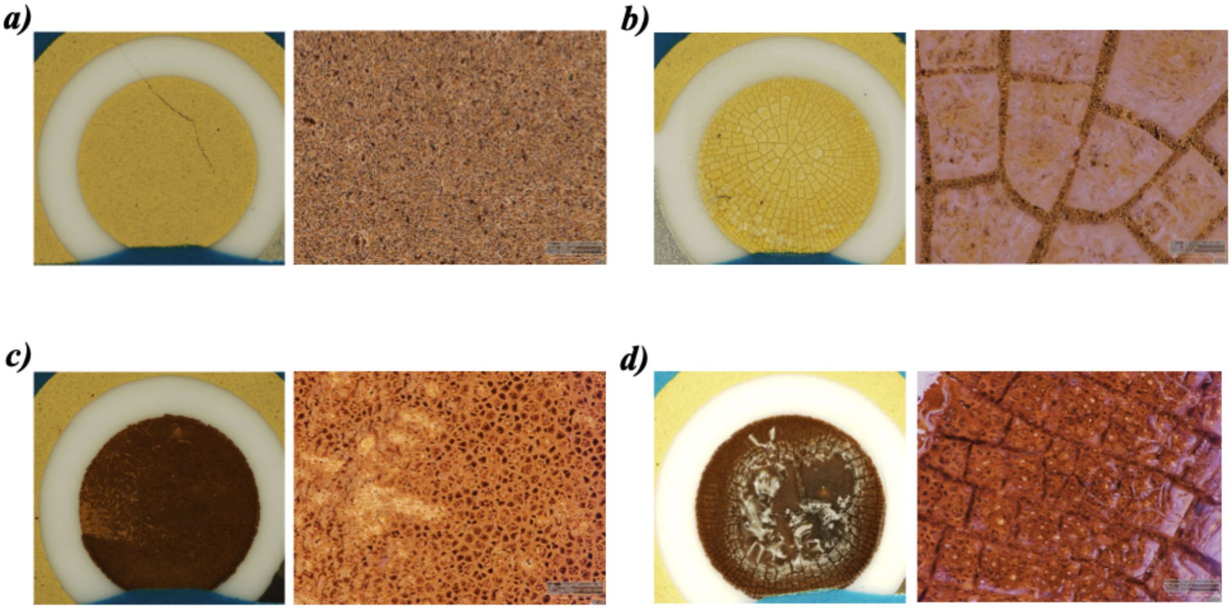

Figure S3. Digital microscopy images of working electrode surfaces: AuSPE before (a) and after (b) BSA adding at 32 mg/mL for 30 min; h-PG/AuSPE before (c) and after (d) BSA adding at 32 mg/mL for 30 min. Images on the left and right (a, b, c, d) were obtained at two different magnifications.
